# A High Energy Density Azobenzene/Graphene Oxide Hybrid with Weak Nonbonding Interactions for Solar Thermal Storage

**DOI:** 10.1038/s41598-019-41563-w

**Published:** 2019-03-26

**Authors:** Wenhui Pang, Jijun Xue, Hua Pang

**Affiliations:** 1 National Joint Engineering Laboratory of optical conversion materials and technology, School of Physical Science and Technology, Lanzhou University, Lanzhou, 730000 China; 2 Key Laboratory for Magnetism and Magnetic Materials of the Ministry of Education, School of Physical Science and Technology, Lanzhou University, Lanzhou, 730000 China

## Abstract

Incorporating photochromic chromophores into polymer composites provides the possibility of a reversible photoswitch of the intrinsic properties of these materials. In this paper we report a route to attach azobenzene (AZO) moiety covalently to graphene oxide (GO) to create chromophore/graphene oxide (AZO-GO) hybrid, in which GO is both part of the chromophore and the template. Due to the high grafting density of AZO moiety and the low mass of the novel structure, the hybrid is a potential solar thermal storage material with high energy density of about 240 Wh·kg^−1^. It is found that C-H···π interaction between the *cis*-AZO chromophores and the aromatic rings of the substrate induces collective electronic modifications of GO at critical percentage of *cis*-isomers and reduce the thermal barrier of π-π* transition of the chromophores directly, which results in two sections of first-order reactions during the photoisomerization of *trans*- to *cis*-hybrid and also thermally stabilizes the *cis*-hybrid. Our findings demonstrate that high-performance AZO–GO hybrid can be manipulated by optimizing intermolecular nonbonding interactions.

## Introduction

In the last decades, numerous efforts have been focused on photo-responsive metastable materials because of their high potential capability for various applications from photorefractive^1^, electro-optics^2^, and energy storage to photodetectors^3,4^. The azobenzene (AZO) and its derivatives can undergo a *trans* → *cis* isomerization under ultraviolet (UV) light. The reverse *cis* → *trans* isomerization can be driven by light or occurs thermally in the dark. In recent years, the photochromatic properties remotivate research interest in AZO based hybrids, as “light-gated” transistors^5^, photochromic molecular switches^6^ as well as solar thermal storages^7–11^. As to the last field, to increase the energy density and storage lifetime, an important strategy is to covalently link AZO photoisomers to polymer substrate, such as carbon nanotube (CNT) templates^12^, reduced graphene oxide (RGO)^5^ and other carbon-based templates^13^, to form orderly arranged molecules. It is reported that the energy densities of some AZO-nanostructure hybrids are comparable to that of Li-ion batteries^8,11^. Evidence has accumulated that the storage capacity and stability of the hybrids can be improved by manipulating intermolecular interactions and inter-planar bundling interactions^8,9,11,12^. For example, remarkable increase in Δ*H* (the enthalpy difference between the *cis*- and *trans*- isomers) can be obtained by optimizing inter and intra molecular H-bonds^7^. In addition, the density functional theory calculations show that the interactions between π-electrons of neighboring phenyl rings of *trans*-AZO molecules with proper intermolecular separation cause a net increase of 0.2 eV in Δ*H* per molecule^3^.

**Figure 1:**
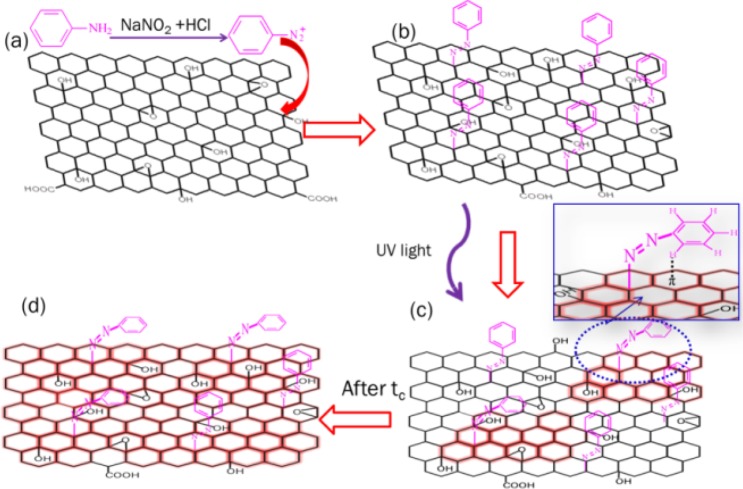
The synthesis route (**a**), chemical structures of *trans* AZO-GO hybrid (**b**) and *cis* AZO-GO hybrid (**c**,**d**).

Like graphene, GO has a two-dimensional sp^2^-hybridized network with π-electrons delocalized over the rings^14^. Moreover, GO is a kind of functionalized graphene, which incorporates carboxylic, hydroxyl and carbonyl groups at its edges, and epoxy and hydroxyl groups on its basal plane^15^. On the other side, aromatic AZO can be formed by a coupling reaction between a diazonium salt and a coupling agent. In the coupling reaction, the benzene diazonium salt behaves as a weak electrophile and attacks carbon atoms with high electron cloud density in phenol ring, such as hydroxyl (-OH) *para*- or *ortho*-carbon sites. Considering that the edges and surfaces of GO are coated with -OH groups, it is reasonable to adopt GO as a coupling agent to yield AZO-GO hybrid containing a nitrogen atom in the diazo salt covalently bonded to a carbon atom in the aromatic ring of GO (Fig. 1). Consequently, GO is both part of the chromophore and the template in the hybrid.

In this paper AZO-GO hybrid was synthesized and the photoisomerization process was investigated. The hybrid is proved to be a potential solar thermal storage material and the high energy density can be ascribed to the high-density grafting of the AZO moiety and the novel structure of the hybrid. Here we demonstrate that, except for classical hydrogen bond, the weak C-H···π non-bonding interaction, appearing in a series of compounds bearing C-H and π-electron systems^16,17^, is important in tuning the thermodynamic and kinetic parameters of AZO-GO hybrid. The mechanism of the kinetics during the *trans-*to-*cis* isomerization of the hybrid has been attributed to the effect of C-H···π interactions between *cis*-AZO chromophores and the aromatic rings of the substrate, which also leads to impressive thermal stability of the *cis*-hybrid.

## Materials and Methods

### Materials

Graphite was obtained from Qingdao Tianhe graphite Co. Ltd. Aniline (AR), Sodium nitrite (AR), ammonia (AR), and ammonium chloride (AR) were obtained from Tianjin bo di chemical co LTD. Anhydrous ethanol and hydrochloric acid were obtained from the hanlon bower (Tianjin) pharmaceutical chemical co LTD.

GO was obtained by using modified Hummers method through oxidation of flake graphite^18^. RGO was prepared by chemically reducing GO using NaBH_4_. 0.1 g, GO was dispersed and reduced in 30 mL of NaBH_4_ (0.1 g) solution (pH = 9) at 80 °C for 1 h. After rinsing and filtration with DI-water, the products were centrifuged for 5 min followed by drying in vacuum at 40 °C overnight.

### Synthesis of AZO-GO hybrids

Firstly 10 mg GO, exfoliated by ultrasonication in 10 ml deionized water to attain an aqueous dispersion, was slowly added to ammonia and ammonium chloride buffer solution (90 mL, pH = 9) and was kept in an ice bath at 0–5 °C. Typically, 6 mL sodium nitrite (0.69 g, 10 mmol) solution was slowly added to the solution of aniline (0.93 g, 10 mmol) in hydrochloric acid (6 M, 10 mL) through a dropping funnel in 10 min. The solution was kept under strong magnetic stirring for 20 min. The solution of diazonium salt was slowly added to GO buffer solution at 0–5 °C with stirring for 0.5 h, then was kept at room temperature for 3 h. The resulting suspension was filtrated and washed with DI-water until the pH of the filtrate reached 6–7, followed by drying under vacuum at 40 °C overnight.

### Characterization

Fourier transform infrared spectroscopy (FT-IR) spectra were recorded on NEXUS-670 spectrometer with a disc of KBr. X-ray photoelectron spectroscopy (XPS) analyses were performed with a Kratos Axis Ultra DLD on model surface analysis system with a 450 W Mg Kα X-ray (1000–1500 eV) source at a base pressure in the 10^−8^ to 10^−9^ Torre range. Thermogravimetry-differential analyses (TGA) of the samples were conducted in air condition by using a STA PT1600 simultaneous thermal analyzer (Linseis Germany). The energy density of AZO-GO was evaluated by differential scanning calorimetry (DSC) on the same apparatus with the heating rate of 5 °C/min during the temperature range of 20 °C to 180 °C. The X-ray diffraction (XRD) patterns were taken by an X′Pert Pro X-ray diffractometer of Philips using Cu Kα radiation (λ = 0.15 nm) at a voltage of 40 kV and a current of 40 mA. UV–vis absorption spectra were recorded at room temperature in ethyl alcohol solution on a UV–vis spectrophotometer (LG-722SP). Time evolutions of the absorption spectra of the AZO-GO and AZO-RGO hybrids in ethyl alcohol solution (1 × 10^−4^ g/mL) upon irradiation of UV light at 365 nm were performed by Perkin Elmer950 spectrometer at room temperature. Raman spectra were recorded by J.Y.HR800, all sample powders were measured excited at 532 nm.

## Results

The interlayer effect and the crystallization of pristine GO and RGO were analyzed by XRD spectra (Fig. S1(a)) which showed that GO and RGO were well dispersed before functionalization with AZO^19^. According to the FT-IR spectra (Fig. S1(b)), the oxygen functional groups attached to the GO and RGO surfaces mainly composed of hydroxyl (-OH), epoxy and alkoxy (C-O-C), and carboxylic (-COOH) groups. The IR band intensities corresponding to the oxygen-containing functional groups were much weaker for RGO due to chemical reduction.

**Figure 2:**
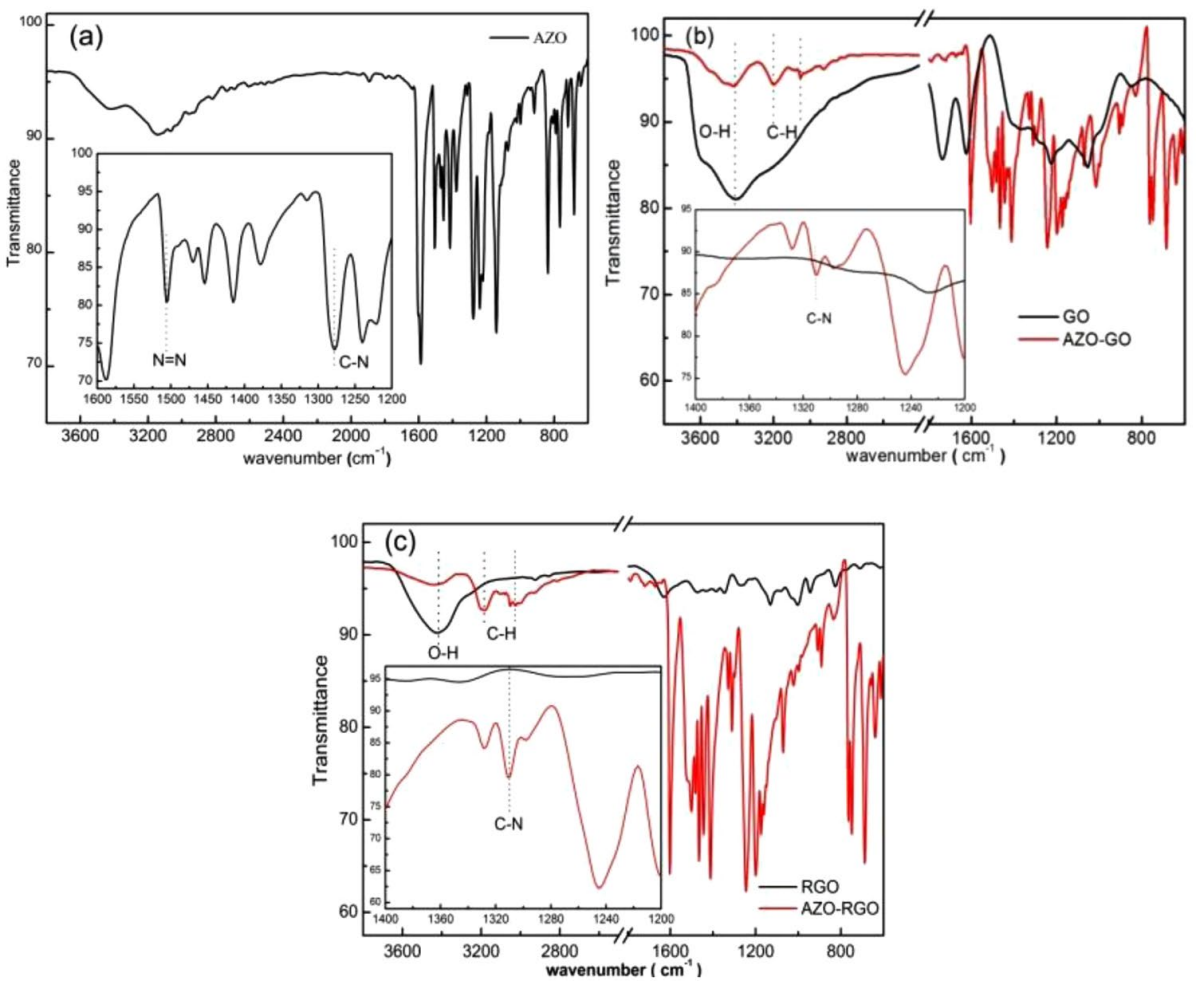
FT-IR spectra of AZO (**a**), AZO-GO (**b**) and AZO-RGO (**c**) hybrids.

The covalent linkage between AZO and GO (RGO) surface can be demonstrated by FT-IR spectra (Fig. 2). For both hybrids, the significant bands are those due to the aromatic ring, AZO chromophore (-N=N-), C-N stretching and other bands, e.g. C-H, etc. The C-H in plane vibration peaks are between 1200–1000 cm^−1^, and out-of-plane vibration of C-H are between 910–665 cm^−1^. The bands due to the aromatic region are in the range of 1400–1600 cm^−1^. The absorption band of the AZO chromophore overlapped with that of the C=C stretching hence a broad band is observed around 1500 cm^−1^ (Fig. 2(a)). Compared with GO and AZO, the absorption of C-N is distinct from the C=C absorbing bands and its frequency ranges centered at 1312 cm^−1^ because resonance increases the bond order between the ring and the attached nitrogen atom (inset in Fig. 2(b,c))^20^. The appearance of C-N bonds indicates the covalent bonding of AZO to GO surface. For the hybrids, another distinguish character happens in the region between 3200 and 3700 cm^−1^, where the intense broad peak corresponding to the -OH stretching vibration in GO is replaced by several distinct peaks. The peaks at 3201 and 3025 cm^−1^ are C-H stretching vibration, which will be discussed at a later stage.

**Figure 3:**
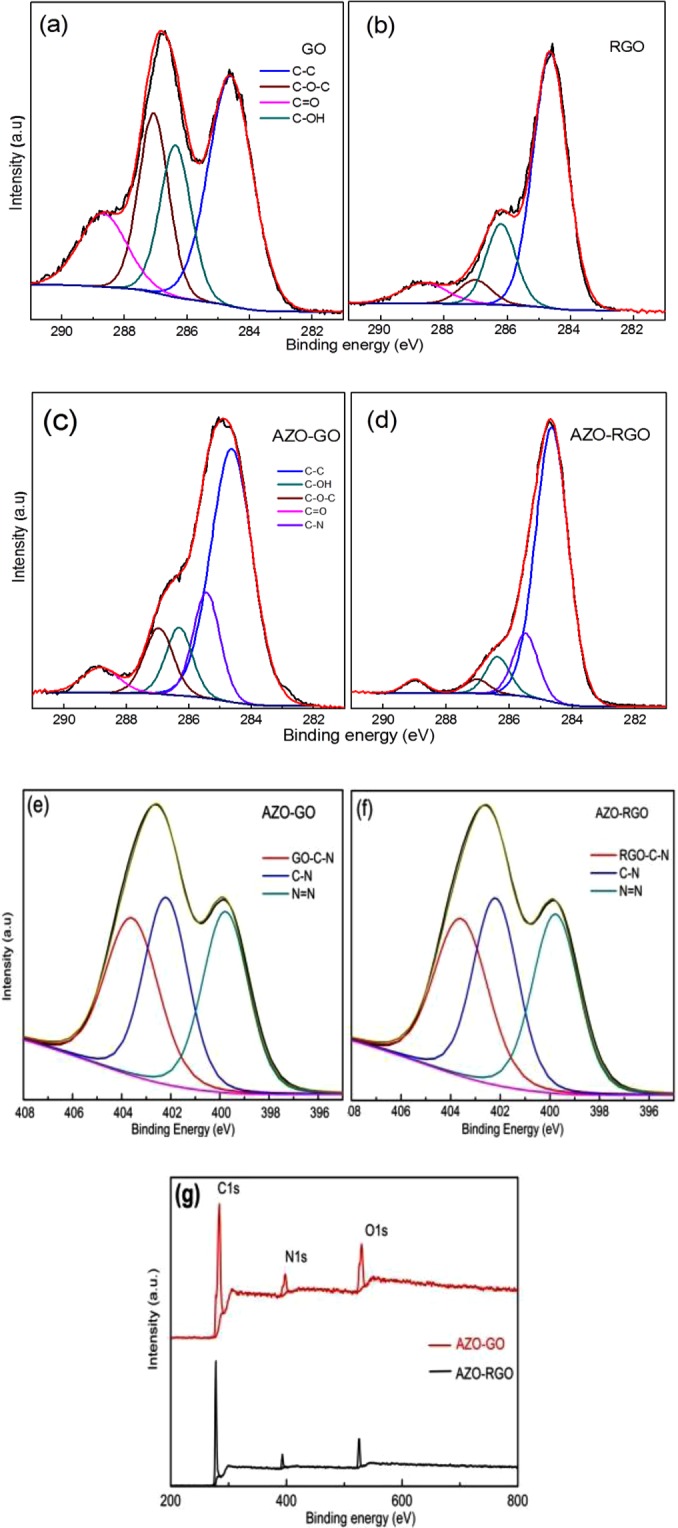
The C 1s XPS and the deconvolution of GO (**a**), RGO (**b**), AZO-GO (**c**) and AZO-RGO (**d**) powder samples. The XPS spectra analysis of N1s for AZO-GO (**e**) and AZO-RGO (**f**). The spectra survey scan (**g**) of AZO-GO and AZO-RGO.

In order to further confirm the covalent linkage between AZO and GO (RGO), X-ray photoelectron spectroscopy (XPS) was used to investigate the chemical structure and composition of GO, RGO, AZO-GO and AZO-RGO powder samples. The deconvolution of each C 1s spectrum has been summarized in Table 1. According to XPS spectrum, it is found that the C/O ratio is 2.96 for GO, indicating a high oxygen atomic percentage in the as-prepared GO though it is a high-bound estimate for the real value^19,21^. The C 1s core level XPS of GO (Fig. 3(a)) shows a peak at 284.6 eV, corresponding to *sp*^2^ carbon framework in a conjugated honey-comb. Three components centered at 286.4 eV, 287.1 eV and 288.7 eV are related to C-OH, C-O-C (epoxy and alkoxy), and C=O groups, respectively, which are in good agreement with the previous reports^21,22^. For RGO (Fig. 3(b)), as listed in Table 1, the intensities of C 1s peaks, especially the peaks assigned to C–O (epoxy and alkoxy) and C=O, decrease dramatically due to the reduction of oxygen-containing functional groups, which confirms the XRD and FT-IR examinations. But the relative content of C-OH hardly changes. As shown in Table 1, the composition percentage of C-OH bond is 19.8% in GO and 18.7% in RGO. The slight difference in binding energy of each bond compared with that in GO can be attributed to the different chemical environment which it is in.

**Table 1:** XPS data of GO, RGO, AZO-GO and AZO-RGO.

Bond		GO	RGO	AZO-GO	AZO-RGO
C-C	Peak BE (eV)	284.6	284.6	284.6	284.6
C=C	At.%	42.8	67.8	58.6	74.4
C-N	Peak BE (eV)	—	—	285.4	285.5
	At.%	—	—	15.9	12.8
C-OH	Peak BE (eV)	286.4	286.2	286.3	286.4
	At.%	19.8	18.7	9.1	7.4
C-O-C	Peak BE (eV)	287.1	287	287	287
	At.%	22.7	6.1	10	2.8
C=O	Peak BE (eV)	288.7	288.6	288.8	288.8
	At.%	14.7	7.4	6.4	2.6
N=N	Peak BE (eV)	—	—	399.0	399.7
	At.%	—	—	33.7	33.2
sp^3^ C-N	Peak BE (eV)	—	—	402.3,	402.2,
	At.%	—	—	32.1	33.3
sp^2^ C-N	Peak BE (eV)	—	—	403.8	403.5
	At.%	—	—	34.2	33.5

The N atoms in AZO-GO and AZO-RGO hybrids may form three kinds of bonds, the N=N bond and C-N bond in the AZO moiety, and the C-N bond connecting AZO to GO (RGO) substrate. The key difference between the two C-N bonds is that the C in the aromatic ring of AZO is at *sp*^2^ state, and the one in GO is at *sp*^3^ state, which has been well proved by the N 1s XPS spectra of the two hybrids (Fig. 3(e,f)). The N 1s spectra of AZO–GO can be decomposed into three components, the one located at 399.0 eV is attributed to N=N bonding, the other two peaks with higher binding energies locate at 402.3 eV and 403.8 eV, respectively, being attributed to *sp*^3^ and *sp*^2^ C-N bonding. According to the experimental data (Table 1), obviously the percentages of the three components are nearly the same, which proves the covalent linkage between AZO and the substrate. As to AZO-RGO, the situation is quite similar except the slight changes of the peak positions.

Figure 3(c,d) display the C 1s XPS of AZO-GO and AZO-RGO hybrids. From the deconvoluted spectra, it is found that in addition to the four aforementioned components in GO and RGO, there is a new one centered around 285.4 eV for the two hybrids, which can be associated with *sp*^3^ type carbon due to the formation of C-N bond. The appearance of C-N bond provides evidence for the covalent linkage between -N=N- group and GO (RGO) substrate. Note that the percentage of C-N is 15.9% in AZO-GO, comparable to that of 12.8% in AZO-RGO. This observation supports our suggestion that the -N=N- group covalently attaches to the aromatic ring with –OH group in GO (RGO) via a diazo coupling reaction (Fig. 1). According to the elemental composition and the atomic percentage of the emerging nitrogen from XPS, as shown in Fig. 3(e), the N/C ratio is 0.089 for AZO-GO, indicating a functionalization density of one AZO moiety for every sixteen GO carbon atoms (noted as 1/16). As to AZO-RGO, the N/C ratio is 0.067 and the functionalization density is 1/24. That is, the average nearest distance between two AZO moieties is ~3.76 Å (~4.26 Å) in AZO-GO (AZO-RGO), which is comparable to previous studies^9^.

**Figure 4:**
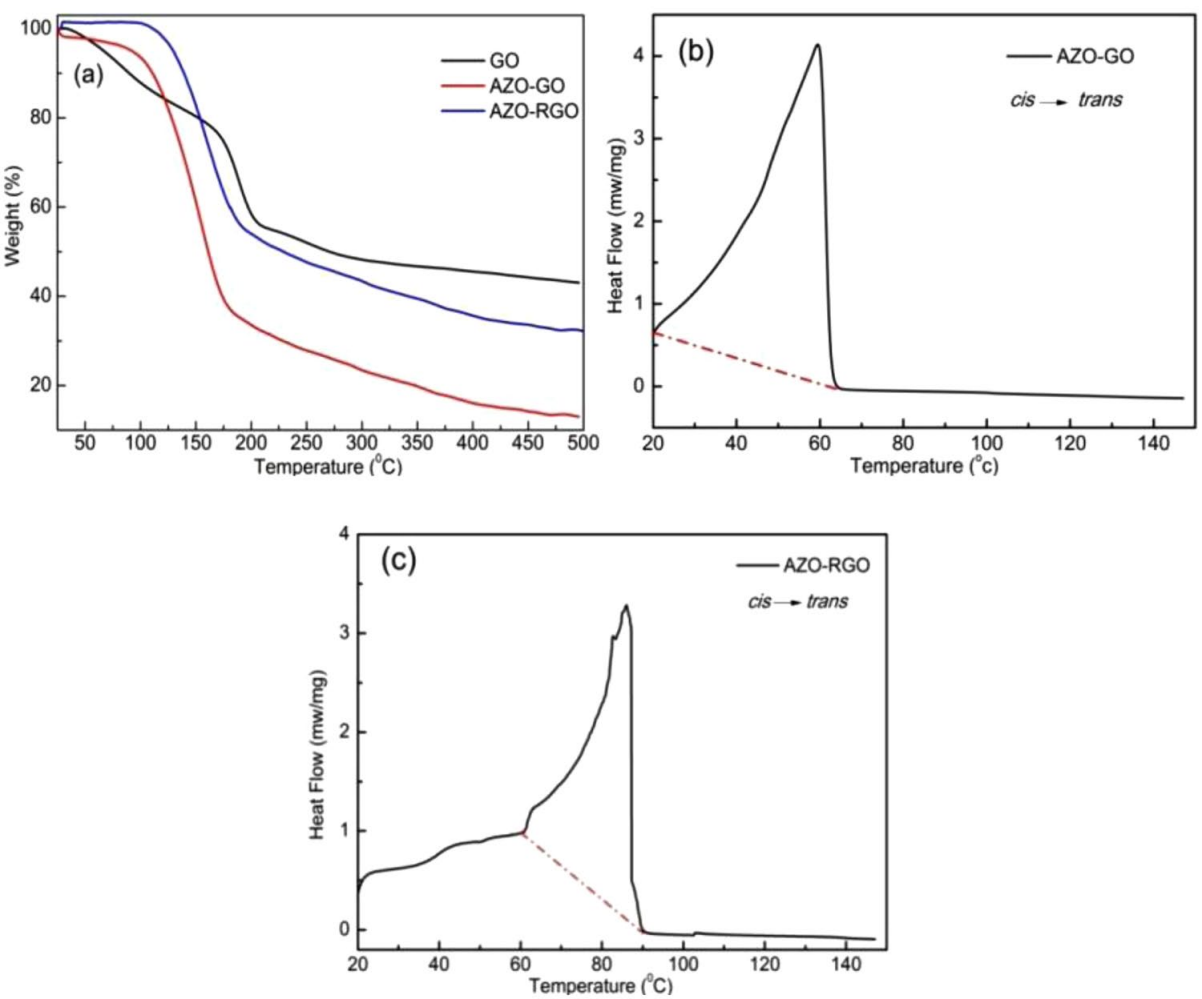
(**a**) TGA of GO, AZO-GO and AZO-RGO hybrids; (**b**,**c**) Integration of the exothermic heat flow corresponding to *cis* → *trans* isomerization of AZO-GO and AZO-RGO.

The amounts of AZO moiety functionalized on GO and RGO were also estimated by TGA (Fig. 4(a)). The TGA weight loss curve of GO shows two steps of weight losses in the temperature region of 25–500 °C. A mass loss of about 25% can be seen at 175 °C, which is resulted by the evaporation of physically adsorbed water on the GO surface. A sharper mass loss happens hereafter and the mass loss of about 45% can be seen at 200 °C, which is assigned to the burning of labile oxygen containing functional groups, yielding CO and CO_2_ as by-products^23,24^. While the AZO-GO hybrid exhibits a steady weight loss from room temperature to 100 °C where the mass loss is about 7%, then the curve drops sharply till 175 °C, where the mass loss is about 65%. Thermal decomposition temperatures of AZO-RGO is slightly increased incomparison with AZO-GO. AZO-RGOis thermally stable from 25 °C to 111 °C, and subsequently shows a sharp weight loss of 44.7% between 111 °C and 175 °C followed by slow loss from 175 °C to 500 °C^25^. Obviously, both hybrids show higher functionalization degrees than GO, confirming the covalent attachment of AZO molecules on GO and RGO surfaces. The grafting degree was calculated to be 38% (20.6%) from the weight losses of the AZO-GO (AZO-RGO) assemblies at different heating stages using Equation 1 in ref.^8^, from which the functionalization density is approximately 1/15 (1/25) (Equation S1 in ref.^9^), agrees well with the XPS measurements.

**Figure 5:**
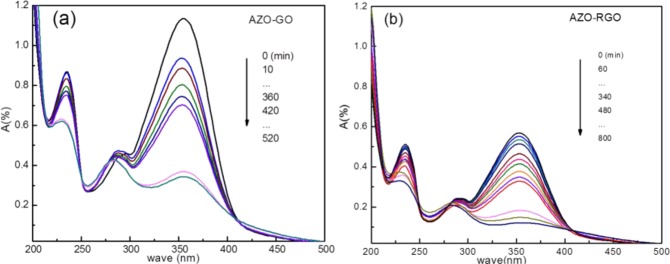
Optical modulated UV–vis absorption spectral changes of (**a**) AZO–RGO and (**b**) AZO-GO in ethyl alcohol upon UV illumination at 365 nm (measured at room temperature 20 °C).

The room temperature photoisomerization properties of AZO-GO and AZO-RGO hybrids are investigated by UV–vis spectra^7,26^. Typical time evolutions of the absorption spectra of the AZO-GO and AZO-RGO hybrids in ethyl alcohol upon UV light irradiation at 365 nm are shown in Fig. 5(a,b). It is well known that for -N=N- group, the two lowest electronic transitions of isolated *trans*-AZO are the ππ* band due to the S_0_ → S_2_ transition centered at 320 nm and the nπ* band due to S_0_ → S_1_ transition centered at 440 nm^26^. Accordingly, the strong band peaked at 354 nm (λ_max_) in the UV–vis spectra of AZO-GO and AZO-RGO hybrids characterize the π–π* transition, while the red shifts compared with that in isolated AZO originate from electronic interactions between AZO and GO (RGO). The weak absorption band centered at 280 nm is the n–π* transition and the strong band at 234 nm is related to the π-π* transition of the GO substrate^15,18^.

Photoisomerization of AZO-GO (Fig. 5(a)) and AZO-RGO (Fig. 5(b)) hybrids can be well indicated by continuous decrease in the band intensity at 354 nm owing to the transformation from *trans*- to *cis*-isomers with the increasing irradiation time. A photostationary state between the *trans*- and *cis*- isomers is established after 800 minutes irradiation, much shorter than that of AZO/graphene hybrid^7^. Hereinafter the *trans*-hybrid after irradiating under UV light for 800 minutes at room temperature is called *cis*-hybrid for convenience. The isomerization degree for *cis*-hybrid, R, can be evaluated from the relation: R = (A_0_ − A_∞_)/A_0_ × 100, where A_0_ is the initial absorbance and A_∞_ is the absorbance at the photostationary state, which yields *R* = 68.9 wt%. This result is comparable to previous reports about dense packing AZO hybrids^8,27^. An interesting observation is that the main UV absorbing band red shifted from 354 nm to 358 nm after radiation longer than 360 minutes, while the center of n–π* transition of GO substrate blue shifted for about 6 nm at the same time. The reasons will be discussed latter.

**Figure 6:**
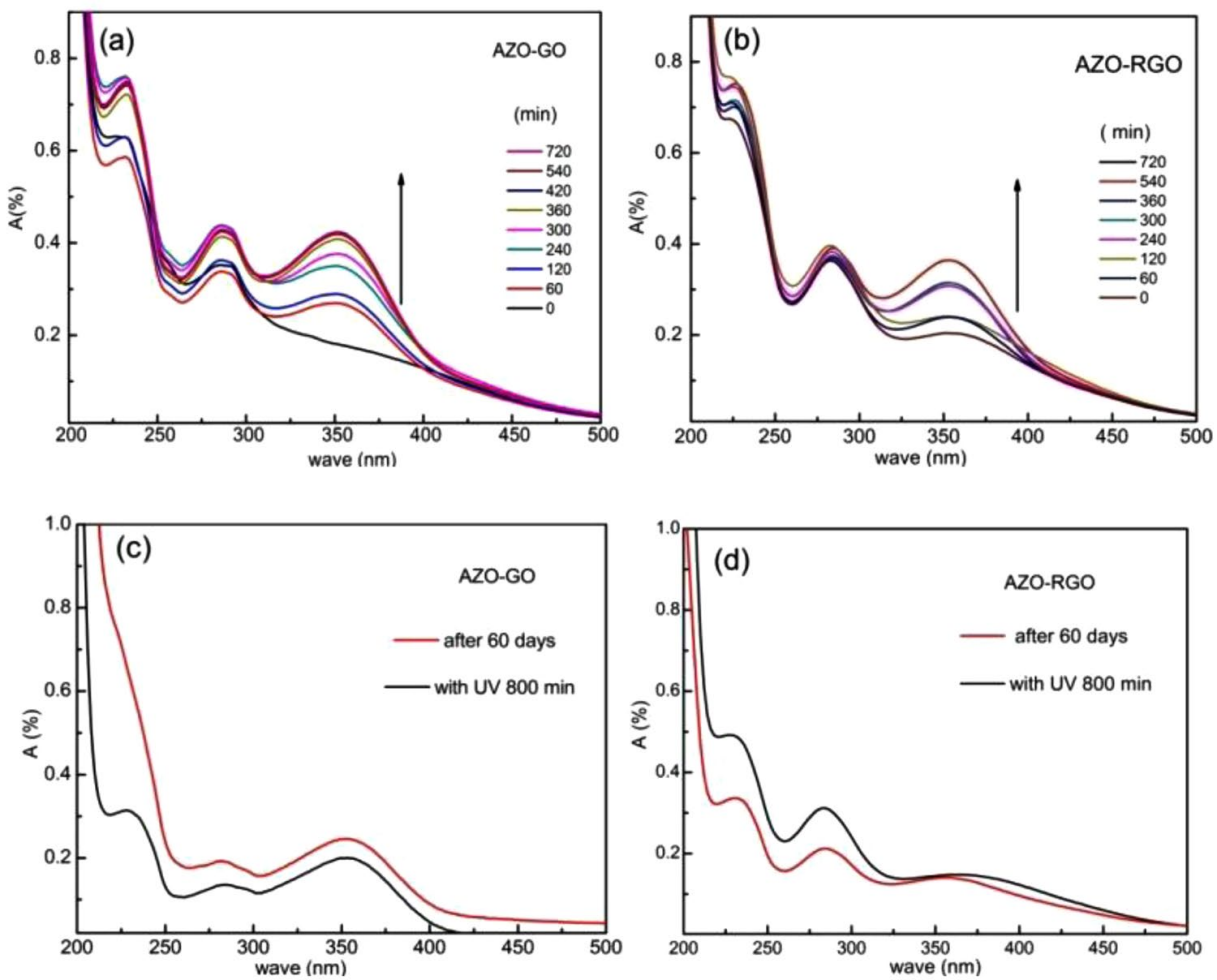
The reversibility and rome stability of AZO-GO (**a**,**c**) and AZO-RGO (**b**,**d**).

Reversion of *cis*- to *trans*- isomerization of both hybrids was studied using UV-vis absorption spectra. The *cis*-hybrids were irradiated under violet (400–430 nm) at room temperature. The results are shown in Fig. 6(a,b). As can be seen, the intensity of the main band centered at 354 nm increases with time, which proves the reversibility of the *trans*- to *cis*- photoisomerization of the hybrids. Thermal reversion of *cis*-hybrid was also studied by UV absorption spectra. The *cis*-hybrid samples were kept in darkness (in a transparent glass) for 60 days before the measurements. As shown in Fig. 6(c,d), the band in the region 320–450 nm in the UV spectrum is rather weak for each hybrid, indicating good thermal stability of the *cis*-hybrid.

The energy density of the AZO-GO and AZO-RGO hybrids were determined by DSC analyses. As shown in Fig. 4(b), the exothermic heat flow of *cis*-hybrid due to *cis* → *trans* isomerization is over 20–65 °C, which corresponds to a bulk gravimetric energy density of 240 Wh·kg^−1^ (199 Wh·kg^−1^ for AZO-RGO). Obviously, higher grafting density results in higher energy density. The energy density is much higher than previous reports about similar hybrids^7–10^, it is even larger than soft-packing Li-ion batteries (90–150 Wh·kg^−1^)^28^. Accounting for the *cis*- and *trans*- compositions of the photostationary state from XPS, the enthalpy difference ∆H between the *cis-* and *trans-* isomers was calculated to be 173.7 kJ·mol^−1^ (114.2 kJ·mol^−1^ for AZO-RGO), which is also outstanding compared with reported materials^7,9^. The high grafting density of AZO moiety in the hybrid is one reason for the improved energy density. The capacity of thermal storage is another key factor to decide the energy density. First principles calculations of ΔH for per AZO moiety is performed based on density functional theory (DFT). The model is that one AZO moiety is supported by 24 carbon atoms of RGO. (Details of the calculations are presented in the supporting information.) Using the relaxed steric structures of *trans*- and *cis*-isomers (Fig. S4), the calculated ΔH is about 0.86 eV/f.u., much larger than the value of ~0.56 eV of an unsubstituted AZO molecule in gas phase^3,7^. More importantly, due to the novel structure, where GO is not only the template but also acts as a component of the chromophore, the hybrid has much smaller molar mass than the other materials, which increase the energy density per unit weight greatly.

**Figure 7:**
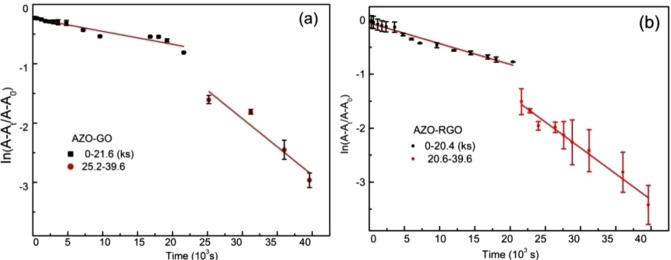
The kinetics constants for *trans→cis* photoisomerization.

To investigate the kinetics of the *trans–cis* photoisomerization of the hybrids, the rate constant and the activation barrier Δ*E*_a_ (energy required for isomerization of per *trans*-AZO moiety) were studied. Previous studies demonstrate that the *trans*-to-*cis* isomerization of AZO in the presence of UV light follows first-order kinetics^29,30^ and the time dependence of the rate constant κ can be written as follows:

1 $$\mathrm{ln}\,\frac{{A}_{\infty }-{A}_{t}}{{A}_{\infty }-{A}_{0}}=-\,\kappa t$$

where *A*_0_, *A*_*t*_ and *A*_∞_ are the absorbance before irradiation, at irradiation time *t* and after irradiation for a prolonged time. Three times of independent UV–vis absorption measurements have been performed using hybrids synthesized in different batches, the average value of $\mathrm{ln}\,\frac{{A}_{\infty }-{A}_{t}}{{A}_{\infty }-{A}_{o}}$ for each hybrid is plotted as the function of *t* in Fig. 7.

Interestingly, for each hybrid, the $\mathrm{ln}\,\frac{{A}_{\infty }-{A}_{t}}{{A}_{\infty }-{A}_{o}}-t$ curve has two discrete segments and for each

segment the curve behaves linearly. The discontinuity happens at about *t*_*c*_ = 360 min (340 min) for AZO-GO (AZO-RGO), after that the curve drops much steeper with time. Note that for both AZO-GO and AZO-RGO hybrids, the proportion of *cis*-hybrid is about 60% at *t*_c_. The rate constant *κ* can be deduced from the slope, which yields κ_1_ = (1.93 ± 0.19) × 10^−5^ s^−1^ when 0 < *t* < *t*_*c*_, κ_2_ = (9.51 ± 1.93) × 10^−5^ s^−1^ when *t*_*c*_ < *t* < *t*_∞_ for AZO-GO. As to AZO-RGO, (9.51 ± 1.93) × 10^−5^ s^−1^ and κ_2_ = (9.61 ± 0.58) × 10^−5^ s^−1^. Interestingly, the kinetics of *cis-*to-*trans* isomerization also shows deviation from the first order kinetics (Fig. S3 for details).

The fact that there exists two segments of kinetic processes during the *trans*-to-*cis* isomerization for both hybrids is quite different from previous reports where the rate constant *κ* is unique during the experimental time limit. For a first order reaction, according to the Arrhenius equation, $\kappa =A{e}^{-\Delta Ea/RT}$ (where *A* is frequency factor), the rate constant is kept still only for cases where the reaction conditions are fixed and only the concentrations of the reactants change with time. In the present case, the temperature is not changed and *A* can be taken as constant also. So the changes in *κ* can be reasonable ascribed to the variation of Δ*E*_*a*_^7^, which can be estimated as

2 $${\rm{\Delta }}{E}_{a}=-\,kT\cdot \,\mathrm{ln}\,\frac{h\,\mathrm{ln}\,2}{{\tau }_{1/2}kT}$$

where *τ*_1/2_ is the time required for half of the *trans*-hybrids to transform to *cis* version and *τ*_1/2_ = ln2/κ, *T* is the temperature, *k*_B_, *R* and *h* are the Boltzmann constant, gas constant and Plank constants, respectively.

As shown in Table 2, τ^1^_1/2_ = 10 h for AZO-GO samples stimulated less than *t*_c_, while *τ*^2^_1/2_ = 2 h for those under irradiation for times longer than *t*_c_. Applying equation (2) and the calculated *τ*_1/2_, we obtained the Δ*E*_*a*_ = 1.02 eV (0.96 eV) for 0 < *t* < *t*_*c*_ (*t*_*c*_ < *t* < *t*_*∞*_). Clearly, after *t*_*c*_ the isomerization energy barrier is lower and the process proceeds faster. The calculations match with the observed red shift of the π–π* transition band in UV–vis absorption spectral after *t*_c_.

**Table 2:** Kinetics Constant (*κ*) and thermal barrier (Δ*E*_*a*_) of AZO-GO and AZO-RGO hybrids. (*t*_*c*_ = 360 min (340 min) for GO (RGO)).

	0 < *t* < *t*_*c*_			*t*_∞_ > *t* > *t*_*c*_		
	κ_1_	${{\boldsymbol{\tau }}}_{{\bf{1}}{\boldsymbol{/}}{\bf{2}}}^{{\bf{1}}}$	Δ*E*_*a*_ (eV)	κ_2_	${{\boldsymbol{\tau }}}_{{\bf{1}}{\boldsymbol{/}}{\bf{2}}}^{{\bf{2}}}$	Δ*E*_*a*_ (eV)
	(×10^−5^ s^−1^)	(h)		(×10^−5^ s^−1^)	(h)	
AZO-GO	1.93 ± 0.19	10	1.02	9.51 ± 0.19	2	0.96
AZO-RGO	3.84 ± 0.19	5	0.98	9.61 ± 0.58	2	0.96

The lower activation barrier of AZO-RGO (Δ*E*_*a*_ = 0.98 eV) than that of AZO-GO before *t*_*c*_ is related to the lower density of AZO moiety in AZO-RGO. The *trans*-to-*cis* isomerization is influenced by steric hindrance and strong intermolecular interaction between AZO units. In the AZO-RGO hybrids, the lower AZO density results in larger separation distance of neighboring AZO molecules and weaker intermolecular interaction, which reduces the steric hindrance, hence lowers the *trans* to *cis* activation barrier. After *t*_c_, the content of *cis*-isomers has exceeded the *trans*-isomers in both hybrids. Consequently, the distance between the neighboring *trans*-isomers increases and the intermolecular interaction decreases, which attenuates the difference in steric effect to the *trans-*to-*cis* isomerization in the two hybrids. Then the reaction barriers are mainly dominated by the electronic structures of the -N=N- group and the two hybrids have the same Δ*E*_*a*_ after *t*_c_.

**Figure 8:**
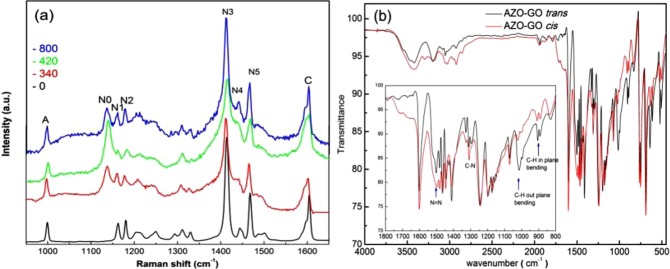
(**a**) Raman spectra (excited at 532 nm) of AZO-GO hybrid after exposure to UV (365 nm) for different times. (**b**) FT-IR spectra of *trans*-AZO-GO and *cis*-AZO-GO hybrids.

To investigate the mechanism of the two-stage first-order reaction kinetics of *trans-*to-*cis* photoisomerization of the hybrid, we recorded a series of Raman spectra, which can be used to elucidate the *in situ* properties of molecules containing the AZO chromophore^31^. Figure 8(a) shows the results of AZO-GO samples after exposed under UV (365 nm) light for different times, the assignments are given in Table 3.

**Table 3:** Raman peak area ratio of AZO-RGO at different time under UV light radiation.

Modes	A	N0	N1	N2	N3	N5	C
(cm^−1^)	998	1137	1163	1180	1414	1468	1605
0 min	0.11	0	0.13	0.12	1.00	0.32	0.46
340 min	0.27	0.64	0.26	0.33	1.00	0.37	0.36
420 min	0.05	0.48	0.09	0.11	1.00	0.39	0.75
800 min	0.07	0.33	0.19	0.17	1.00	0.36	0.53

A strong Raman signal was obtained for the as prepared *trans*-hybrid. The Raman active mode located at about 999 cm^−1^, indexed as Mode A, is ascribed to the aromatic rings breathing. Mode N1 and N2 centered at 1165 cm^−1^ and 1180 cm^−1^, respectively, are predominately C-N symmetrical stretches coupled with C-H in-plane bending contributions of the AZO phenyl ring^31,32^. The three bands in the -N=N- stretching region, 1400–1465 cm^−1^, are named N3, N4 and N5. The band at about 1600 cm^−1^, named as Mode C, involves the stretching of the aromatic ring. The bands in the region of 1200–1260 cm^−1^ can be attributed to the combination of the C-C stretching and the C-H in-plane bending, the band in the region 1280–1340 cm^−1^ can be ascribed to the conjugation between the oxygen containing groups and the phenyl ring^31^. These two bands are less important in probing the dynamics in photoisomerization process hence were elided in the following. Contributions from other molecular constituents are much less pronounced. It is found that, compared with the pristine GO and RGO (Fig. S2), the G and D band of graphene are absent in Fig. 8(a). This can be understood as the result of proton transfer from AZO to GO during chemisorbing, which inducing blue shift of G band and significant decrease of I_D_/I_G_^33^. The blue shift of G band makes it overlap with the C band of AZO, while the D band is masked by the N3 band of AZO, which is broad and strong.

Obviously, Raman scattering of the *trans*-hybrid is the strongest for modes which elongate the molecule along its length causing a change in polarization of the molecule^31,34^. After exposing under UV light, the samples have composition of *cis*-isomers, which results in weaker spectrum due to the reduced symmetry. In the meanwhile, a new C-N mode appears at about 1136 cm^−1^, named N0. Band N0 requires population of excited-state S_1_^35^. In *trans*-hybrid the intense contributions of ground state S_0_ to the Raman Spectrum may hinder the isolation signals from S_1_ hence N0 is absent. For irradiated samples, the appearance of N0 can be considered as an important signature of isomerization.

To get further insight of the temporal structural information of the hybrids under UV light before and after *t*_*c*_, we calculate the intensity of each mode compared with that of Mode N3, the strongest peak for all cases, at specific exposing time and show the results in Table 3. Due to the weak intensity and strong overlapping with N3 after irradiation, it is hard to differentiate the variation tendency of Mode N4 hence not shown. As shown in Table 3, the intensity ratio of Mode N5 is nearly constant till the exposing time limit, while the relative intensity of Mode A, N0, N1, N2 decreases suddenly after exposing for *t*_*c*_, meanwhile, the intensity of Mode C increases abruptly. Clearly, distinct intensity anomaly appears for modes related to the vibrations of the aromatic rings after *t*_*c*_. Simultaneously, the peak position of the characteristic -N=N- stretching, Mode N4, shifts toward lower frequency side for 4 cm^−1^ immediately after *t*_c_. While in *cis*-hybrid, all modes undergo red shift compared with *trans*-hybrid, for example, Mode N1 and N2 are shifted by about 3.0 cm^−1^, mode A and mode C are shifted by about 2 cm^−1^.

The anomalies in intensities and positions of these modes before and after *t*_*c*_ represent the abrupt variations of the vibration modes after exposing under UV light for *t*_c_. Comparative analysis of the infrared spectra of *trans*- and *cis*-hybrid provide further evidences. As shown in Fig. 8(b), for both hybrids, the significant bands corresponding to the aromatic ring, AZO chromophore (-N=N-), C-N stretching are similar in positions and profiles, the increased intensity of C-N and N=N modes in *cis*-hybrid is reasonably ascribed to the reduced symmetry of *cis*-hybrid. However great changes take place for C-H modes. The C-H in-plane bending mode and C-H out-of-plane bending mode are in the region 900~1100 cm^−1^ and 650–900 cm^−1^, respectively (inset of Fig. 8(b)), while the C–H stretching vibration modes are observed in region 3000–3100 cm^−1^. As can be seen, for *cis*-hybrid, the phenyl ring’s C–H stretching vibration is enhanced while the C-H bending bands are strongly prohibited compared with *trans*-hybrid. This observation indicates a vertical or a tilted orientation of the AZO phenyl ring to the substrate for *trans*-isomer whereas the AZO moiety is more parallel to the GO substrate for *cis*-isomer.

## Discussion

With use of the Cambridge Structure Database, Yoji Umezawa and coworkers put forward a method for exploring X-H···π interaction^36^. According to this method, whether there is a C-H···π interaction in AZO-GO hybrid depends mainly on two factors, the first is the distance *d* between H atom and the aromatic ring underneath, the second is the angle of *θ* (=∠HC_AZO_C_GO1_, C_AZO_ is the carbon atom on the AZO moiety which connected directly with the H atom, C_GO1_ is the nearest carbon neighbor of H atom on the aromatic ring of GO). It is found that C-H···π interaction happens given appropriate parameters, i.e., *d*_*max*_ < 3.05 Å and *θ* < 60°. Based on the calculated bond lengths and bond angles of AZO moiety in ref.^37^, and set the dihedral angle between AZO aromatic ring and GO surface as 30° (90°) for *cis*-isomer (*trans*- isomer), it is found that the *d*_*cis*_ = 3.01 Å for *cis*-isomer, while *d*_*trans*_ = 3.82 Å for *trans*-isomer. Obviously, *d*_*trans*_ is much larger than *d*_*max*_, which means the C-H···π interaction cannot occur in *trans*-isomer while it is possible in *cis*-isomer. Moreover, the energy difference in Δ*E*_*a*_ of AZO-GO before and after *t*_c_ is about 1.38 Kcal/mol (0.06 eV), matching with the typical energy of C-H···π bonds^38^.

According to this assumption, the intensity and Raman shift mentioned above can be well explained. As shown in the inset of Fig. 8(b), the intensities of C-H stretching mode are enhanced in *cis*-hybrid, while the intensity of C-H bending mode reduces. It is believed that the coupling of C-N vibration with C-H in plane bending strengthens the band intensity of C-N in Raman spectra^32^. The formation of C-H···π stacking hinders the C-H in-plane bending and reduces the coupling with C-N related modes hence weaken the band intensity. Similarly, coupling of C-H stretching with the aromatic ring increase the polarizability of C=C stretching mode and increase the intensity of Raman Mode C, at the same time the ring breathing mode is limited hence reducing the band intensity of Mode A.

We would ascribe the origin of the two segments of linear dynamics during the *trans*-to-*cis* isomerization as a direct consequence of the existence of C-H···π interaction. When a *cis*-isomer comes into being, the C-H···π hydrogen bond makes the H atom of the AZO benzene moiety act like an electron donor, while the aromatic ring of the GO substrate beneath acts like an acceptor, which results in local electron-rich region in the GO surface^38^. The perturbations to the electronic structures of GO will enhance the polarizability of GO substrate. The area of electron-rich region grows with the extension of irradiation time. When there are enough percentage amount of *cis*-isomers, about 60% at *t*_c_, the area of electron-rich region links up and the perturbation is strong enough to modify the electronic distributions of the GO substrate collectively, which influence the electronic structure of *trans*-hybrid and *cis*-hybrid directly. For simplicity, the effect of increasing the electron-rich region is analogy to increasing the polarity of the solvent, which decreases (increases) the energy gap between π-π^*^(n-π^*^) states. As depicted in Fig. 5, the π-π^*^ transition of -N=N- group in *trans*-hybrid red shifted while the n-π^*^ transition of GO blue shifted after *t*_c_. The energy gap reduction of π-π^*^ also leads to red shift of Mode N4 in Raman spectra and decreased activation energy Δ*E*_*a*_ during the *trans*-to-*cis* isomerization, which also contributes to the thermal-stabilization of *cis*-hybrid. The red shifts of other Raman active modes in *cis*-hybrid indicate coupling of the -N=N- stretching to the C=C and C-H modes of the aromatic rings.

Our observations prove that, though C-H···π interaction is a weak nonbonding interaction, it is capable to tune the band structures of GO. A recent theoretic calculation reports that a band gap of ~90 meV is opened by C-H···π interaction for graphene^14^. The modifications of the band structures originate from the broken of inversion symmetry of the two carbon sub-lattices of graphene due to heterogeneous distributions of C-H groups. So it is inferred that the key factors to decide *t*_c_ are the grafting density of AZO and the spatial uniformity of C-H groups above GO, as well as the intensity of the C-H···π interaction. Therefore, besides changing the functionalization degree of the hybrid by modifying preparation method, such as multiple iterating reaction^8^, substituting partial H atoms of the AZO aromatic ring by -XH groups (such as -NH_2_, -OH, -CH_3_, etc.) can influence *t*_c_ directly^39^. Substituting induces asymmetric distribution of nonbonding interactions, what’s more, the substituent can have a dramatic impact on XH···π interactions, which depends strongly on the nature of the group^14^.

Now there are two key problems towards commercial applications of AZO-based solar thermal fuels. One is to increase the energy storage density and the second is to reach a suitable thermal barrierΔ*E* for the *cis* → *trans* back reaction. Dense molecular packing of the covalently attached AZO molecules on carbon substrates can effectively increase the bulk energy density, as have been proved here and in refs^3,4,7,29^. More importantly, it provides possibility to systematically control the inter- or intra-molecular interactions in the hybrids^7–10^. However single mechanism usually improves in Δ*H* at the cost of decreasing Δ*E*^7^. This work provides a thought to adjust the stability of *cis* and *trans* states independently hence to decouple and increase both the energy density and thermal barrier of the hybrids since C-H···π interaction happens only between the *cis*-AZO moiety and the GO substrate due to the peculiar structure of AZO-GO hybrid. To improve the stability of the *trans*-hybrid, the intermolecular hydrogen bond can be introduced by proper substituent of the AZO phenyl ring. Other adjustable parameters include molecular assembly density, chemical composition and position of the joining groups, etc.

## Conclusion

AZO-GO (RGO) hybrid is prepared by diazotization method. XPS and FT-IR results prove that the AZO monomer is covalently bonded to the GO (RGO) substrate and the functionalization density is approximately 1/16 (1/24) for AZO-GO (AZO-RGO). The UV–vis spectra of AZO-GO hybrid prove reversible photoisomerization in region of 300–400 nm. The novel hybrid exhibits a high energy density up to 240 Wh·kg^−1^ and good thermal stability of *cis*-hybrid. Vibrational spectra of the hybrids highly suggest the existence of C-H···π interaction between the aromatic ring of the AZO to that of the GO matrix in *cis*-isomer, which induces collective electronic modifications of the hybrid and influences the π–π* transition of the chromophores directly. The kinetics of the *trans* to *cis* photoisomerization includes two discrete linear segments. The discontinuity happens at *t*_*c*_ when the percentage of the *cis*-hybrid is about 60%, after that the activation energy drops and the photo isomerization process proceeds faster.

## Supplementary information

A High Energy Density Azobenzene/Graphene Oxide Hybrid with Weak Nonbonding Interactions for Solar Thermal Storage
