# Supplementary material for: A High Energy Density Azobenzene/Graphene Oxide Hybrid with Weak Nonbonding Interactions for Solar Thermal Storage
Source: Sci Rep. 2019 Mar 26;9:5224. doi: 10.1038/s41598-019-41563-w (PMC6435660; doi:10.1038/s41598-019-41563-w)
Supplement: Supplementary file 1 — A High Energy Density Azobenzene/Graphene Oxide Hybrid with Weak Nonbonding Interactions for Solar Thermal Storage [file 41598_2019_41563_MOESM1_ESM.docx]

**Supporting information**

**A High Energy Density Azobenzene/Graphene Oxide Hybrid with Weak Nonbonding Interactions for Solar Thermal Storage Wenhui Pang^1^, Jijun Xue^2^, and Hua Pang^1^***

^1^ National Joint Engineering Laboratory of optical conversion materials and technology, School of Physical Science and Technology, Lanzhou University, 730000, China

^2^ Key Laboratory for Magnetism and Magnetic Materials of the Ministry of Education, School of Physical Science and Technology, Lanzhou University, 730000, China

*Corresponding authors. [hpang@lzu.edu.cn](mailto:hpang@lzu.edu.cn)

^+^these authors contributed equally to this work

**XRD patterns of the pristine GO and RGO**


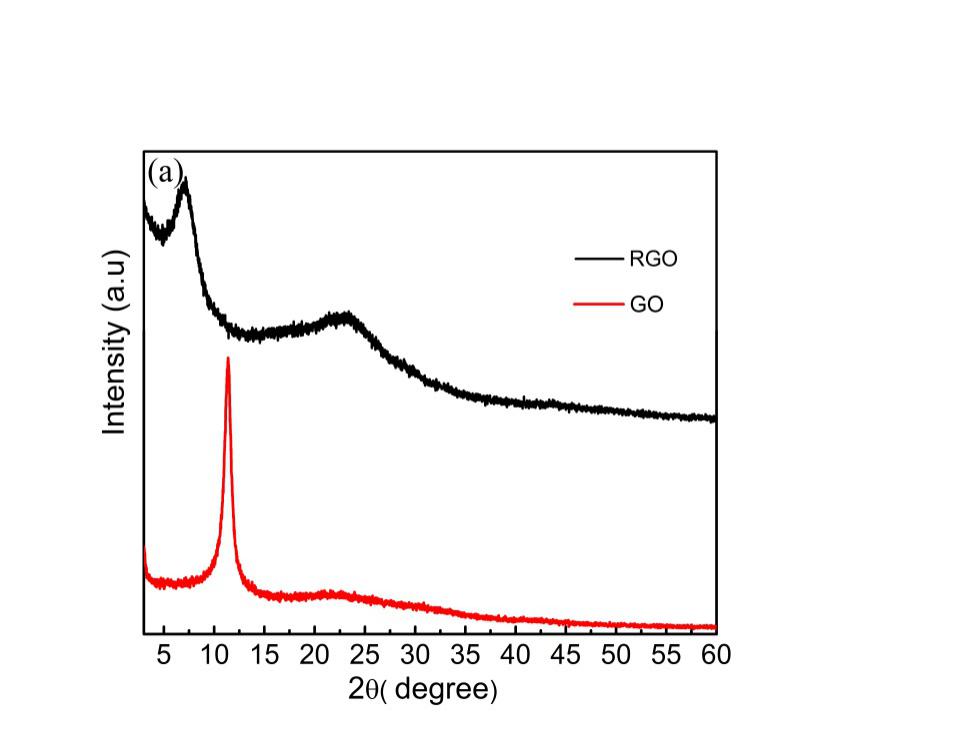

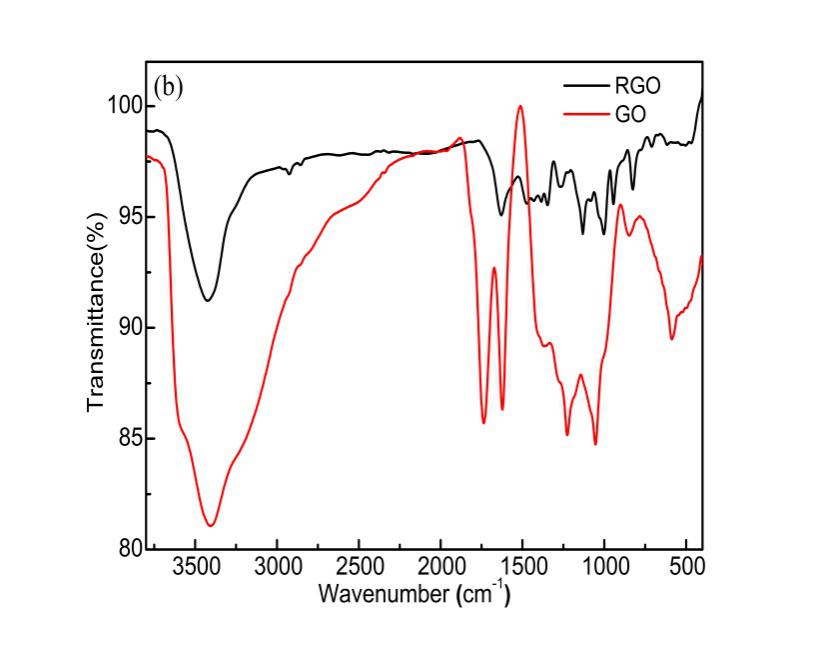


Figure S1 XRD patterns (a) and FT-IR spectra (b) of GO and RGO.

Figure S1 (a) shows the XRD patterns of the pristine GO and RGO. The pattern of GO reveals an intense and sharp peak centered at 2θ=11.5°，corresponding to the (001) interplanar spacing of 0.8 nm. After reduction the parent GO with NaBH_4_, this peak shifts to 7.0° and becomes much weaker, which can be attributed to increased interlayer spacing and disorder. And a new broader peak is centered at 2θ =25.0° corresponding to the *d*-spacing of 0.36 nm, which is more likely to be the (002) peak of graphite.

The oxygen functional groups attached to the GO surface can be well demonstrated by the FT-IR spectra. As shown in Figure S 1 (b), the peak at 1732 cm^−1^ is corresponding to the stretching vibrations of C=O bonds, the peak at 1225 cm^−1^and the one at 1052 cm^−1^ can be assigned to the C-O groups in epoxy and in alkoxy, respectively. Hydroxyl (-OH) groups have an intensive absorption band between 3200 and 3700 cm^−1^, due to the presence of moisture intercalated within hydrophilic GO sheets. The peak at 1575 cm^-1^ is ascribed to the skeletal vibrations of aromatic C=C bond.For RGO, the characteristic bands for C=O bonds almost disappear and the band width of –OH groups get narrower compared with those of GO. While the peak appeared at 1470 cm^−1^can be ascribed to larger π-conjugated C=C structure. These observations indicate that most of the oxygen-containing functional groups were partial restored by NaBH_4_.

**Raman spectra of GO and RGO**


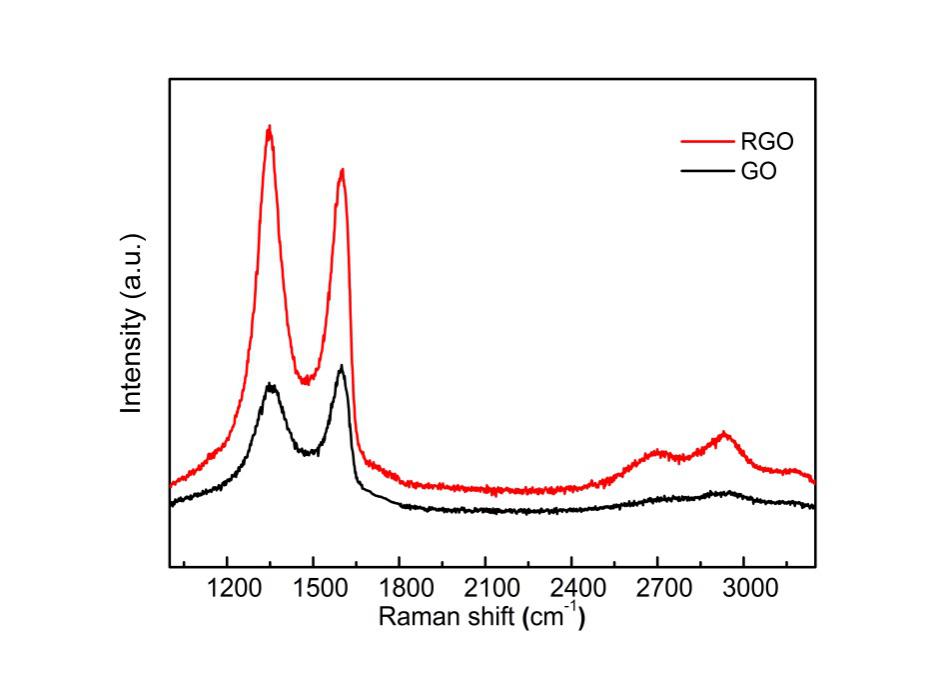


Figure S2 Raman spectra (excited at 532 nm) of GO and RGO.

Figure S2 shows the Raman spectra of GO and RGO. The first-order G and D peaks of GO, both arising from the vibrations of sp^2^ carbon, appear at around 1594 cm^-1^and 1356 cm^-1^, respectively. As compared to the G band of GO, that of RGOwas shifted by 9 cm^-1^ from 1594 to 1603 cm^-1^,displayinga high reduction degree.

**Kinetics of *cis* to*trans*isomerization**


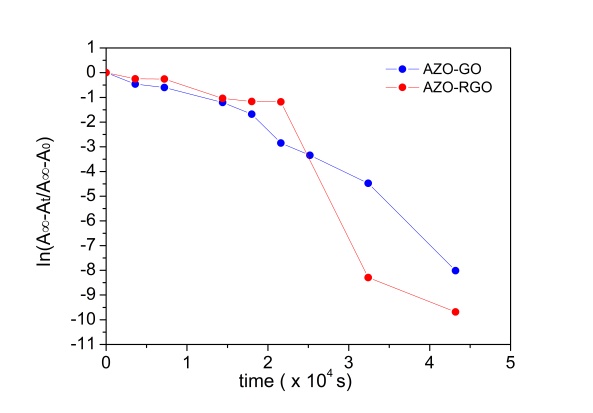


Figure S3Dependenceofln[(A_∞_-A_t_)/(A_∞_-A_0_)] onirradiationtime(at430nm)ofAZO-GO and AZO-RGO hybridsafter irradiation at365nm for 800 mins.

Figure S3 gives the kinetic behaviors of *cis*to*trans*isomerization of the two hybrids under visible light (430nm) irradiation. For each hybrid, the departure from first order kinetics is quite obvious and may be ascribed to the intermolecular interactions between AZO and GOandthemodifications of the electronic structure of AZO due to the novel steric structure of the hybrid.

**First-principles calculations of the structures of *trans*- and *cis*- isomers**

The first-principles calculations were carried out using the Wien2k package.^S1^ The full-potential linearized augmented plane-wave (FLAPW) method based on density functional theory (DFT) were adopted, where the generalized gradient approximation (GGA) (Perdew-Burke-Wang form) was employed for the exchange correlation energy functional. The plane-wave cut off parameter *R*MT ×*K*max was 4.0 and the magnitude of the largest vector *G*max was 14.0. There were 8 k-points in the irreducible Brillouin zone. The model system has 24 nonequivalent carbon atoms and one AZO moiety in a unit cell, corresponding to a functionalization density of 1/24. Periodic boundary conditions were adopted for the XY directions of the unit cell except Z-axis, which was set as 15 Å to avoid interlayer interactions along Z-axis. The volume and lattice parameters of the structures were optimized and the atomic positions in each system were fully relaxed until the force on each atom was smaller than 5.0 mRy/a.u.

The main results of DFT calculations are displayed in Figure S4. The energy of the model system isplotted as the function of the dihedral angle θ between the AZO aromatic ring and the GO substrate (Figure S4 (a)). Obviously, the configuration with the minimum energy is reached when θ=90°, corresponding to *trans*- state of the hybrid, while a meta-stable state appears at θ=30°, corresponding to *cis*- state of the hybrid. This result conforms well with the experiments. According to the calculations, in the optimized *trans*- state (Figure S4 (b)), the bond length of C_GO_-N, N=N, C_GO_-C_GO_, C_AZO_-H are 1.44Å, 1.23 Å, 1.42 Å, 1.08 Å, respectively, the bond angle of C_GO_-N=N is 118.21°. Considering that the bond length of the *trans-* state is only 0.01 Å shorter than that of the *cis*-state, we fix the bond lengths during the *cis*- state calculations, but relax the bond angles. The bond angle of C_G__O_-N=Nthus obtained in the optimized structure of *cis*-isomer is 127.86° (Figure S4 (c)). These results match well with previous reports.^S2^

For our model system, the calculated enthalpy difference ∆Hbetween the *trans*- and *cis*- states is about 0.86 eV/f.u., much larger than the value of ~0.56 eV ofanunsubstituted AZO molecule in gas phase.^7^ It is reported that DFT calculations without considering weak interactions, which is poorly described within DFT, usually results in a 12-17% reduction in ∆H.^3^ Then a rough estimation yields ΔH of 97kJ/mol, which is coincident with the experimental result of 114.2kJ/mol for AZO-RGO.


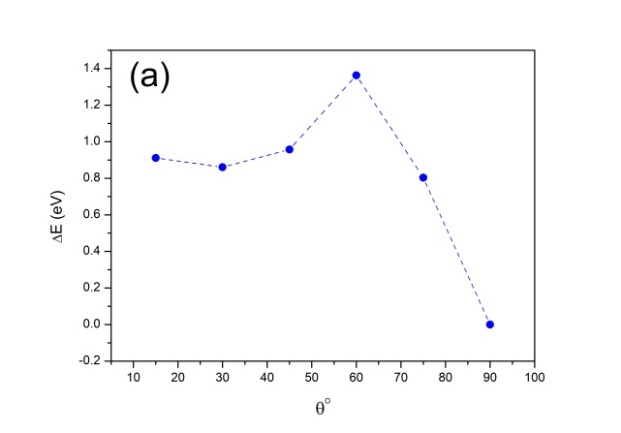


Figure S4(a) The evolution of energy difference Δ*E* (= *E*_θ_- *E*_90°_) with the dihedral angle θ between the AZO aromatic ring and the GO substrate. (b) and (c) are optimized structures of *trans*- and *cis*- states.

**Reference**

S1. Blaha, P.; Schwarz, K., Sorantin, P. & Trickey, S. B.. Full-potential, linearized augmented plane wave programs for crystalline systems. *Computer Physics Communications*, **59**, 399-415 (1990).

S2. Cojocaru, C., Airinei, A., & Fifere, N.. Molecular structure and modeling studies of azobenzene derivatives containing maleimide groups. *SpringerPlus*, **2**, 586 (2013).
